# Prognostic Role of the Activated p-AKT Molecule in Various Hematologic Malignancies and Solid Tumors: A Meta-Analysis

**DOI:** 10.3389/fonc.2020.588200

**Published:** 2020-12-10

**Authors:** Zhen Yao, Guangyu Gao, Jiawen Yang, Yuming Long, Zhenzhen Wang, Wentao Hu, Yulong Liu

**Affiliations:** ^1^ Department of Nuclear Accident Medical Emergency, The Second Affiliated Hospital of Soochow University, Suzhou, China; ^2^ Department of Ultrasound, Xingtang Hospital, Suzhou, China; ^3^ State Key Laboratory of Radiation Medicine and Protection, School of Radiation Medicine and Protection, Soochow University, Suzhou, China; ^4^ Collaborative Innovation Center of Radiological Medicine of Jiangsu Higher Education Institutions, Suzhou, China

**Keywords:** p-AKT, tumor, meta-analysis, oxidative stress, biomarker

## Abstract

Cancer is one of the main causes of human death worldwide. Recently, many studies have firmly established the causal relationship between oxidative stress and cancer initiation and progression. As a key protein in PI3K/Akt signaling pathway, p-AKT (phosphorylated Akt) participates in the process of oxidative stress and plays a prognostic role in various hematologic tumors and solid tumors. We conducted a comprehensive search of the PubMed, Embase and Cochrane libraries to identify studies published in the past decade involving cancer patients expressing p-AKT that reported overall survival (OS) during follow-up. In this study, 6,128 patients in total were evaluated from 29 enrolled articles, and we concluded that overexpression of p-AKT was closely related to worse OS in cancer patients with a hazard ratio (HR) of 2.33 (95% CI: 1.67–4.00). Furthermore, we conducted a subgroup analysis, and the results indicated that overexpression of p-AKT was associated with worse OS in hematological tumor (HR: 1.64, 95% CI: 1.41–1.92), and solid tumor (HR: 2.44, 95% CI: 1.61–5.26). High expression of p-AKT is related to poor prognosis of various hematologic tumors and solid tumors.

## Introduction

In recent years, global cancer cases have shown rapid growth. In 2018, It was estimated that there were 18.1 million new cancer cases and 9.6 million cancer deaths worldwide (1). As a common type of malignant tumor, the incidence of hematological tumors is increasing year by year, and the age of onset is gradually getting younger. Therefore, it is of great necessity to search new biomarkers for early diagnosis of hematologic tumors and solid tumors.

Akt, known as Protein Kinase B (PKB), a key protein in the Akt/PI3K signaling pathway, is a serine/threonine-specific protein kinase which plays an important role in progression of various human cancers and oxidative stress once activated by phosphorylation (2, 3). Furthermore, oxidative stress can trigger damage and modification of cellular macromolecules including genomic DNA, which can produce mutations. Many studies have shown that oxidative damage caused by oxidative stress is an important factor in the formation and development of cancer (4). As a key protein in the Akt/PI3K signaling pathway, p-AKT (phosphorylated Akt) may be an important link between oxidative stress and cancer. Therefore, we researched a large number of studies and found that overexpression of p-AKT is firmly related to the prognosis of various hematologic tumors and solid tumors, such as non-small cell lung cancer (NSCLC) (5–8), gastric cancer (GC) (9–12), squamous cell carcinoma (SCC) (13–16), hepatocellular carcinoma (HCC) (17–19), ovarian cancer (OC) (20, 21), diffuse large B-cell lymphoma (DLBCL) (22, 23), urothelial carcinoma (UC) (24, 25), nasopharyngeal carcinoma (NC) (26), pediatric Burkitt lymphoma (PBL) (27), hairy cell leukemia (HCL) (28), peripheral T-cell lymphoma (PTCL) (29), anaplastic large cell lymphoma (ALCL) (30), breast cancer (BC) (31), endometrial carcinoma (EC) (32), and pancreatic cancer (PC) (33).

In our article, a meta-analysis was conducted to research the HR of p-AKT expression for OS in patients with different kinds of tumor. We further assessed whether it can be used as a new biomarker and also discussed the relationship between oxidative stress and tumor occurrence and metastasis.

## Materials and Methods

### Literature Search Strategy

Studies published from April, 2010 to April, 2020 were extracted from PubMed, Cochrane Library, and Embase. The following key words were adopted: (p-AKT or phosphatidylinositol-3-kinase/Akt or pl3K-Akt) and (prognosis or prognostic or overall survival or survival rate or disease‐free survival or disease‐free survival) (neoplasia OR neoplasias OR neoplasm OR tumors OR tumor OR cancer OR cancers OR malignancy OR malignancies OR malignant neoplasms OR malignant neoplasm). At the same time, we have also consulted the English version of the list of potential references for research in order to get more relevant studies for data analysis.

### Data Extraction and Management

Eligible studies were selected on the basis of the following criteria: (a) studies on the association between p-AKT expression and OS or DFS or RFS or PFS in humans with cancer; (b) in tumor tissues, p-AKT protein or mRNAs can be detected; (c) studies dividing patients into two groups on the basis of p-AKT expression stage, and (d) the articles recorded HR with 95% CI or HR with 95% CI can be calculated by sufficient statistics. The criteria for exclusion were (a) studies without sufficient statistics to calculate HR with 95% CI or (b) letters, case reports, reviews, clinical trials, conference abstracts, or (c) not *in vivo* researches. Only English language articles were researched in the article. Two investigators (GY and ZY) were independently responsible for extracting statistics from studies that meet our standards. A third investigator will resolve differences when different opinions arose. We have recorded the following information: name of the first author, country, year of publication, tumor type, sample size, study design, cut-off value, follow-up time, measure method and outcome measure. Meanwhile, we also extracted HRs with 95% CI for OS from selected studies. However, if only the Kaplan–Meier curves can be found in these articles, we will use the software named Engauge digitizer version 4.1 to estimate survival statistics (34).

### Quality Assessment

We evaluated the cohort studies using the Newcastle-Ottawa Scale (NOS) (35). If the score of the study is more than six, it would be considered of high quality.

### Statistical Analysis

In our study, we researched the prognostic value of p-AKT expression in patients with different kinds of tumors by estimating the hazard ratio and elated 95% CI between the low expression of tissue p-AKT groups and the high expression of tissue p-AKT groups for OS, DFS, PFS, or RFS. Moreover, the heterogeneity between studies was evaluated with P-value and I^2^. If I^2^ >50%, we would think that there was significant heterogeneity in this article, and a random-effect model wound be used to calculate the total HR. On the contrary, a fixed-effect model would be used if the heterogeneity of articles was moderate (I^2^ ≤50%). All statistical analyses were performed by using normalized data analyzing procedures offered in Stata 14.0 software.

## Results

### Characteristics of Selected Studies

**Figure 1:**
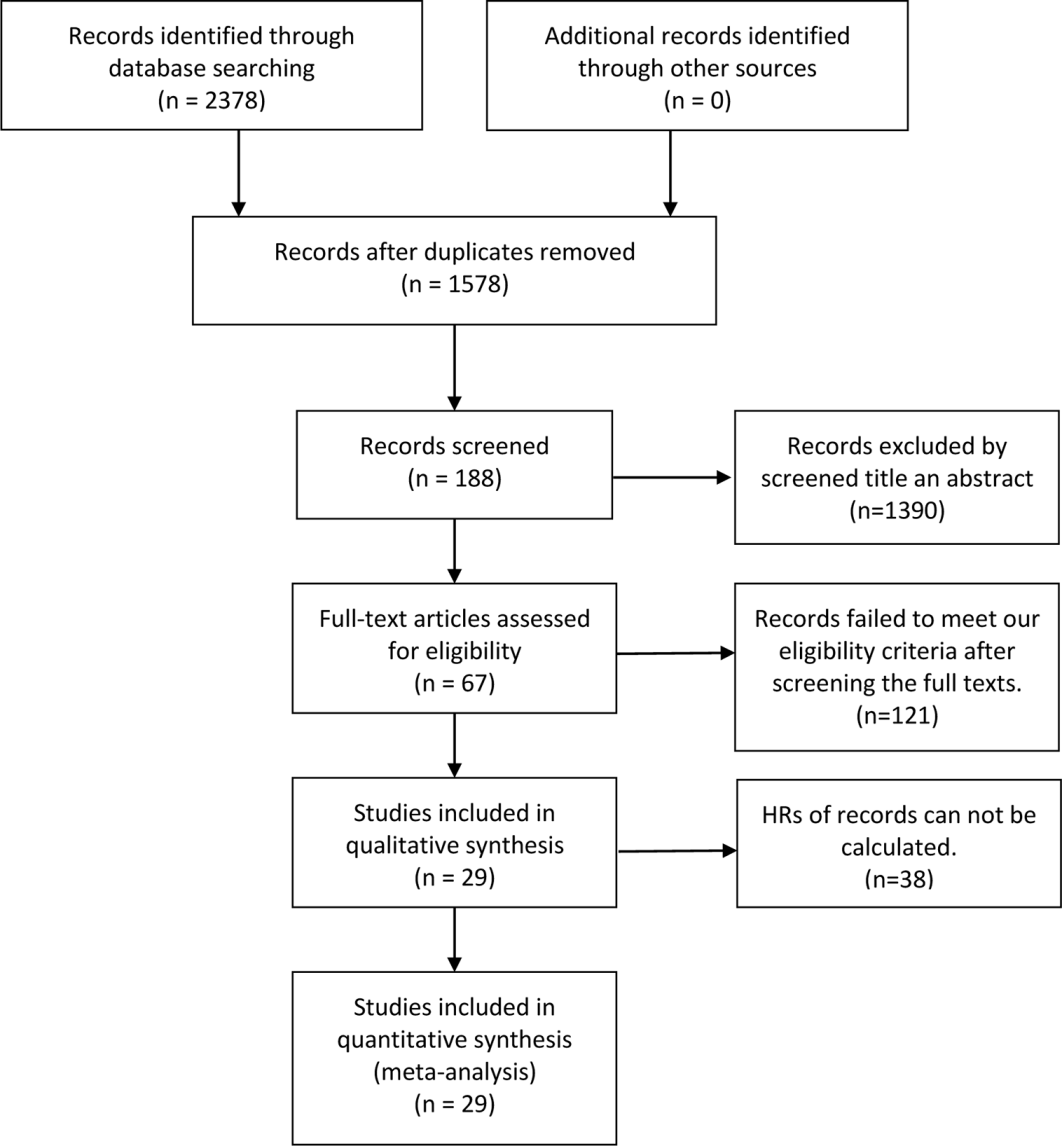
The flow chart of the selection process in our meta-analysis.

The flow chart of the selection process in our study is shown below (**Figure 1**). After searching keywords on the three mentioned databases (Embase, PubMed, Cochrane Library), 2,378 associated articles were identified, then 1,578 articles were viewed after deleting duplicates between databases. After screening the title and abstract, 1,390 articles were removed. Besides, we excluded 121 articles that did not meet our criteria after reading the full text. Of these, 38 records did not calculate the HRs. Finally, 29 studies were identified with 6,128 patients in our study. As revealed in **Table 1**, the sample size of these 29 studies ranged from 43 to 887, with an average of 211.3. Besides, these articles published in the last ten years were from many countries, including 17 in China, four in America, three in Greece, one in Brazil, one in Japan, one in Korea, one in Chile, and one in Sweden. Among these included studies, four were on NSCLC, four on GC, four on SCC, three on HCC, two on OC, two on DLBCL, two on UC, one on NC, PBL, HCL, PTCL, ALCL, BC, EC, and PC. The expression level of p-AKT was determined with immunohistochemistry (IHC) and polymerase chain reaction (PCR) in most articles.

**Table 1:** Characteristics of the selected studies.

Author	Country	Year	Sample	Study design	Sizes	Cut-offvalue	Follow-up time(month)	Outcome	NOS	method	Data
Wang, X.et al. (17)	China	2020	HCC	R	60	25%	36	OS/DFS	6	IHC	E
Man, J.et al. (27)	China	2020	PBL	R	52	20%	60 (24–168)	OS/PFS	7	IHC	C
Lu, J.et al. (5)	China	2020	NSCLC	R	341	25%	0–120	OS	7	IHC	E
Zheng, H. et al. (6)	China	2019	NSCLC	R	247	20%	0–120	OS	7	IHC	E
Li, Q.et al. (13)	China	2019	SCC	R	85	20%	0–120	OS/PFS	8	IHC	E
Feng, J.et al. (26)	China	2019	NC	R	499	25%	0–120	OS	8	IHC	E
Ibrahim, MY.et al. (14)	America	2019	SCC	R	123	25%	44.76 (0–222.36)	OS	9	IHC	E
Azizi, M.et al.	America	2019	SCC	R	57	25%	51.1 (3.1–57.1)	OS	6	IHC	E
Zhang, MH.et al. (18)	China	2018	HCC	R	200	25%	106 (0–207)	OS	7	PCR	E
Wu, N. et al. (16)	China	2018	SCC	R	290	25%	6.88 (0.64–17.05)	OS	8	IHC	E
Chiappini, PBO. et al. (9)	Brazil	2017	GC	R	439	20%	62.4 (32.4–92.4)	OS	8	IHC	C
Liu, W. H.et al. (20)	China	2017	OC	R	43	20%	113.2 (2.3–181.6)	OS	7	IHC	E
Ito, C.et al. (10)	Japan	2017	GC	R	111	25%	160	OS	7	IHC	E
Su, R.et al. (19)	China	2016	HCC	R	208	20%	46 (1–152)	OS	7	IHC	E
Lakiotaki, E.et al. (28)	Greece	2016	HCL	R	77	20%	0–400	OS	8	IHC	E
Ying, J.et al. (11)	Sweden	2015	GC	R	59	25%	82.08 (7.68–204.6)	OS	7	IHC	C
Hong, JY.et al. (29)	Korea	2015	PTCL	R	63	25%	76 (3–167)	OS/PFS	7	IHC	C
jia, W.et al.	China	2014	OC	R	118	25%	3–192	OS	8	IHC	E
Hong, JY.et al. (22)	America	2014	DLBCL	R	262	25%	102.5 (39.6–235.7)	OS	6	IHC	E
Kitano, H.et al. (7)	America	2014	NSCLC	R	220	25%	120	OS	7	IHC	E
Lazaridis, G.et al. (31)	Greece	2014	BC	R	997	20%	192	OS	7	IHC	E
Tapia, O. et al. (12)	Chile	2014	GC	R	142	20%	120	OS	7	IHC	E
Gao, J. et al. (30)	China	2013	ALCL	R	103	25%	0.3–96	OS	6	IHC	E
Xu, ZZ. et al. (23)	China	2013	DLBCL	R	73	20%	72	OS	9	IHC	E
Korkolopoulou, P. et al. (24)	Greece	2012	UC	R	113	20%	120	OS	8	IHC	E
Hu, J. et al. (8)	China	2012	NSCLC	R	114	20%	0–120	OS	7	IHC	E
Yang, X. et al. (32)	China	2011	EC	R	71	20%	120	OS	8	IHC	E
Sun, CH. et al. (25)	China	2011	UC	R	887	25%	0–96	OS	8	IHC	C
Liu, J. Et al. (33)	China	2010	PC	R	74	20%	0–120	OS	8	IHC	E

E, extracted; C, calculated.

### Meta-Analysis

**Figure 2:**
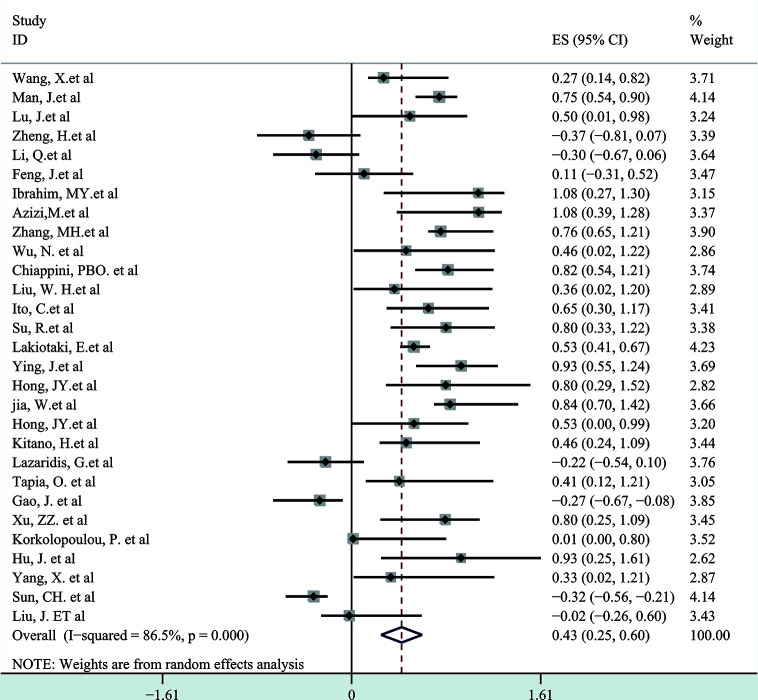
The correlation between p-AKT expression and OS in human tumors.

A meta-analysis was performed to evaluate the relation between expression level of p-AKT and OS of cancer patients. In this research of 29 enrolled articles among 6,128 patients with different kinds of tumor, a significant connection was found between p-AKT expression and OS in patients with tumor (HR: 2.33; 95% CI: 1.67–4.00; p < 0.05; **Figure 2**). Such results may indicate that higher p-AKT expression was related to worse OS in tumor patients. Since significant heterogeneity was discovered (I^2^: 86.5%) among the selected studies that met our criteria, a random-effect model was then used.

**Figure 3:**
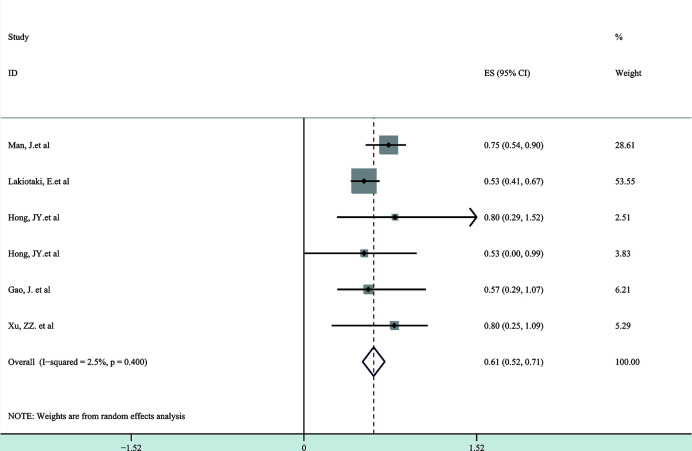
Forest plots demonstrating the relation between p-AKT expression and OS in hematological tumor patients.

**Figure 4:**
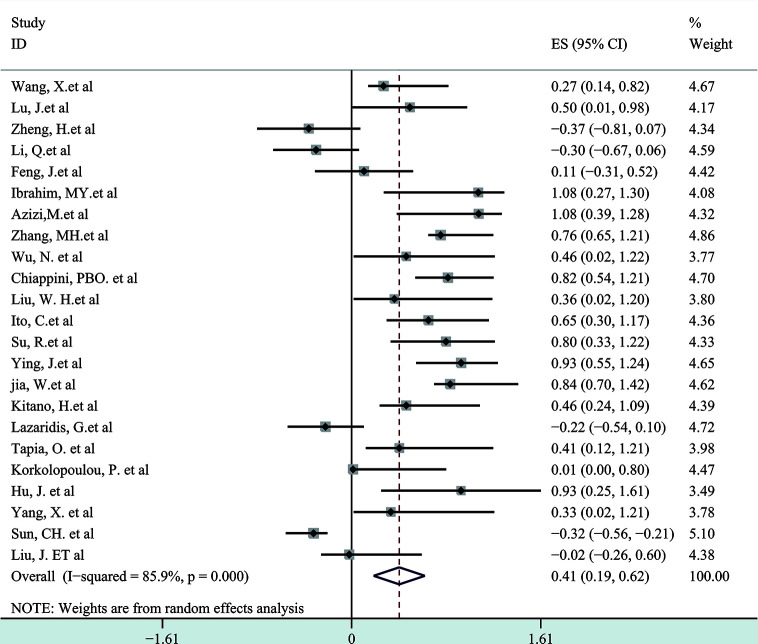
Forest plots demonstrating the relation between p-AKT expression and OS in solid tumor patients.

**Figure 5:**
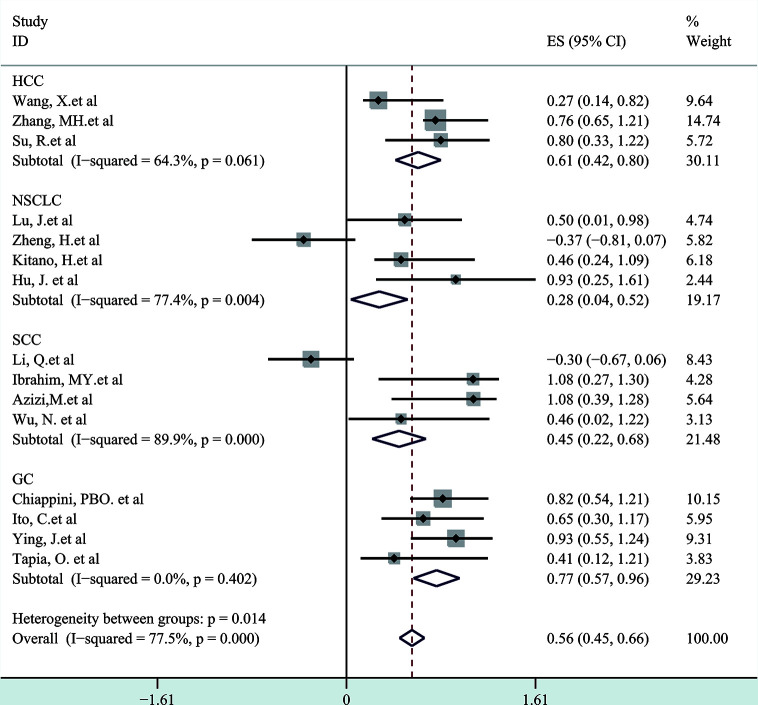
Subgroup analysis of different types of tumors.

**Figure 6:**
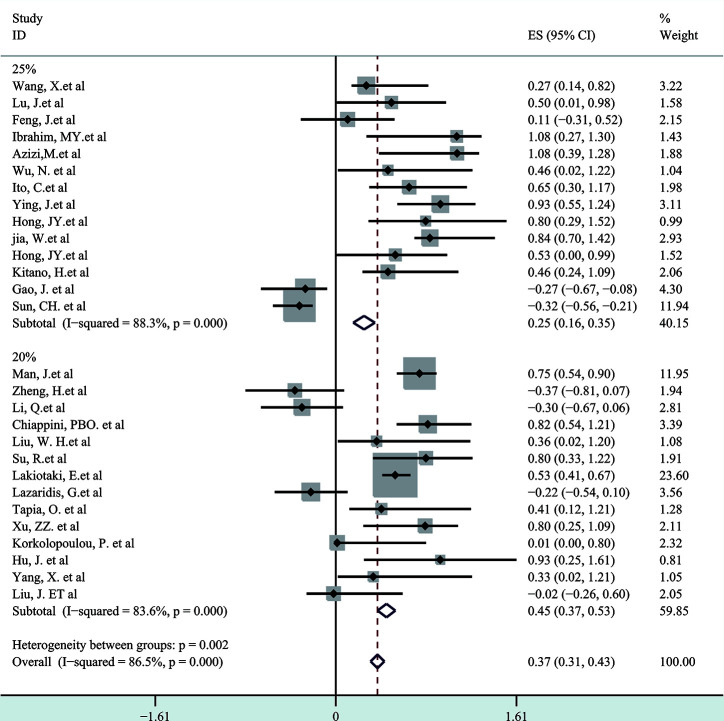
Subgroup analysis of different cut-off values.

Next, a subgroup analysis was carried out to investigate whether heterogeneity is caused by different types of tumors. Tumors were divided into hematological tumors and solid tumors to calculate the total HR (**Figure 3**, **Figure 4**). Obvious heterogeneity was discovered in solid tumors (I^2^: 85.9%). In the hematological tumor subgroup, we didn’t find significant heterogeneity (I^2^: 2.5%). Totally, our results showed that overexpression of p-AKT may cause worse OS in tumors. However, subgroup analysis according to cancer types demonstrated that overexpression of p-AKT may cause worse OS in hematological tumors (HR: 1.64, 95% CI: 1.41–1.92), and more research should be done in solid tumors to prove the relationship between p-AKT expression and OS. Tumor of the same type was also combined to calculate the total HR (**Figure 5**). Moreover, articles about SCC, GC, NSCLC, and HCC were further researched by performing subgroup analysis. Significant heterogeneity was found in SCC (I^2^ = 89.9%), NSCLC (I^2^ = 77.4%), and HCC (I^2^ = 64.3%). After screening all contents, we thought different ethnic backgrounds, sample sizes, and the number of articles were the causes for high heterogeneity, and more researched were needed to verify our results. In the end, we came to the conclusion that positive expression of p-AKT was associated with poor OS in SCC, GC, NSCLC, and HCC. Of all the cited documents, only two articles used PCR (Polymerase Chain Reaction) to evaluate the expression of P-AKT, and the rest used IHC (immunohistochemistry) so we did not consider the heterogeneity of the detection method. According to the cut-off value (20 and 25%), we divided all the literature into two groups. Subgroup analysis was performed. The results showed that both of them approved our conclusions (**Figure 6**).

### Publication Bias

**Figure 7:**
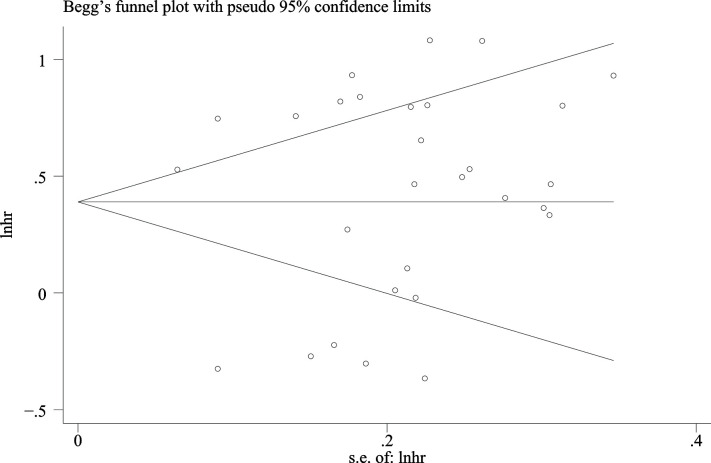
Begg’s funnel plots with 95% CI for publication bias in the included 29 studies.

**Figure 8:**
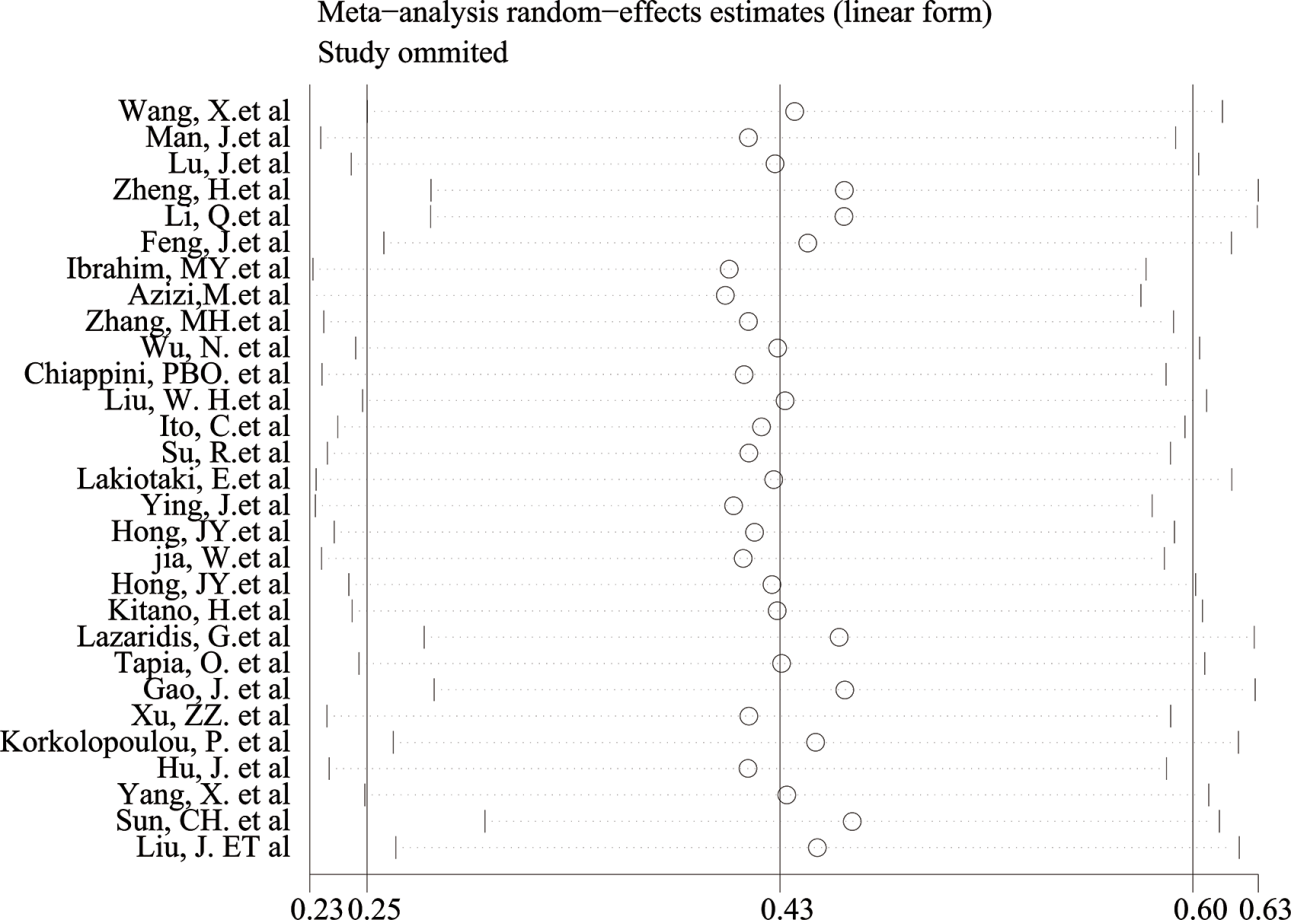
Sensitivity analysis evaluating the impact of individual research on the estimate.

The Begg’s test P-value of 0.675 for OS indicated the lack of significant publication bias (**Figure 7**). Also, a sensitivity analysis was performed to further confirm the credibility of HR for OS. The results showed that there was no significant impact on HR after eliminating any one article, suggesting the reliability of the result (**Figure 8**).

### Discussion

In the past few years, many studies found that the PI3K/Akt signaling pathway plays a vital role in cell differentiation (36, 37). With the activation of the pathway, phospho-Akt (p-Akt) is generated, which may promote cell survival against oxidative stress (38) Moreover, deregulation of the pathway is beneficial for cancer cell survival (including hematological malignancies), promotes chemotherapy resistance through disruption of apoptosis, and initiates cap-dependent translation of mRNAs essential for cell cycle progression, differentiation, and growth. Plenty of researches revealed that both PI3K/Akt signaling pathway and oxidative stress are closely related to the occurrence and development of tumors, and both of them are common features of many human cancers (39). The expansion of cancer is a multi-stage and multi-step complicated process, which involves environmental change and cell gene mutation. Excessive production of reactive oxygen species causes oxidative stress in cells, by which gene mutation can be induced through oxidative adduct formation and/or inhibition of repair enzymes for oxidative DNA damage. In addition, it has been shown that reactive oxygen species (ROS) can influence the expression of important cancer-related genes through second messengers and modification of transcription factors (4).

P-AKT is vital in the process of oxidative stress; what’s more, it plays a prognostic role in various hematologic tumors and solid tumors. Akt is an important protein kinase in a lot of key pathophysiology processes including cell proliferation, angiogenesis, glucose metabolism, apoptosis, cell cycle regulation, oxidative stress, tumor occurrence, and metastasis, which can regulate many downstream effectors once activated by phosphorylation [3], and two activating phosphorylation sites of Akt (T308 and S473) play a key role in the regulation of these cellular functions (40). Related studies have shown that high expression of p-AKT is significantly associated with poor prognosis of patients with NSCLC (41) and GC (42). In addition, through the application of bioinformatics, we found that AKT plays different roles in various specific tumors such as lung cancer, gastric cancer, colorectal cancer, hepatocellular carcinoma, breast cancer, acute myeloid leukemia, *etc.* However, since this systematic review includes many different types of cancers, we cannot conduct further studies on the mechanism of action of AKT in each type of cancer. Among the common pathways of cancer, AKT plays an important role in central carbon metabolism, choline metabolism, PD-L1 expression, and PD-1 checkpoint pathway. To further evaluate the prognostic value of p-AKT in different tumors, we conducted this meta-analysis.

So far as we are aware, the meta-analysis consisting of 29 controlled studies is the only study that has fully researched the published articles on overall survival and p-AKT expression of enrolled tumor patients. Of these 29 articles, 27 have researched the mechanism of the whole PI3K/AKT/mTOR axis. We systematically evaluated the survival data of 6,128 patients with different solid and hematological tumors. Five kinds of hematological tumors and ten kinds of solid tumors were observed, and we came to the conclusion that there was a strong relation between p-AKT overexpression and adverse prognosis of tumors in terms of overall survival (HR: 2.33; 95% CI: 1.67–4.00; p < 0.05). The heterogeneity was high, and subgroup analysis was then performed according to different types of tumor. In the hematological tumor subgroup, we didn’t find any heterogeneity, and the result indicated that overexpression of p-AKT was related to worse prognosis (HR: 1.64, 95% CI: 1.41–1.92; p < 0.05, I^2^: 2.5%). However, the heterogeneity of the solid tumor subgroup remains high (I^2^: 85.9%); we conducted further subgroup analysis of different solid tumors and came to the conclusion that positive expression of p-AKT was related to poor OS in SCC, GC, NSCLC, and HCC. Combined with the sensitivity analysis, publication bias and overall content of the articles, we believed that the sample sizes and the number of studies were the causes for that, and more comprehensive and systematic studies are needed to prove our research.

More and more studies have proved that oxidative stress is important in blood disorders, and p-AKT may be a key protein in the process. In our study, five kinds of hematological tumors including diffuse large B-cell lymphoma, pediatric Burkitt lymphoma, hairy cell leukemia, peripheral T-cell lymphoma, and anaplastic large cell lymphoma were evaluated. The results (HR: 1.64, 95% CI: 1.41–1.92; p < 0.05, I^2^: 2.5%) indicated that overexpression of p-AKT is related to poor prognosis of hematological tumors and may influence prognosis of various blood disorders. P-ATK may become a new target for the treatment of blood diseases.

Our meta-analysis is rigorous and has some advantages. First, we strictly followed the inclusion criteria of the research to ensure the quality of the included research, and the sample that meets the requirements is also sufficient. Second, we strictly picked out related articles from various authoritative databases such as EMBASE, PubMed, and Cochrane Library to ensure the authenticity of the research. Third, we conducted several subgroup analyses to effectively minimize the heterogeneity existing in the qualified studies and to further explore the application potential of p-AKT as a biomarker for prognosis of different kinds of tumors. Fourth, we found that our research has no obvious publication bias through the interpretation of the Begg’s funnel plot. Furthermore, we conducted a detailed and comprehensive literature search to control publication bias. Finally, a methodological evaluation of the research was conducted to avoid selection bias and ensure the comparability and quality of the research.

However, our article also has some shortcomings. First, our article only includes English research. Second, due to the large number of articles, the cutoff point of p-AKT has not been acknowledged, and the cutoff points provided by the original articles were used to evaluate the diagnostic value. Third, only a part of HRs was provided directly, and the rest were calculated by analyzing the survival curve in the selected articles. Thus, the HRs obtained by calculation may not be as precise as the HRs obtained directly from original articles.

## Conclusion

In conclusion, overexpression of p-AKT and the oxidative stress response it participates in may have an intimate relationship with worse overall survival in cancer patients. Especially, over-expression of p-AKT might be a biomarker of worse prognosis in patients with hematological tumors. Also, p-AKT may act as a drug target for tumor treatment in the future.

## Data Availability Statement

The raw data supporting the conclusions of this article will be made available by the authors, without undue reservation.

## Author Contributions

ZY contributed to the design of the project. YLiu contributed to the administrative support. GG, JY, ZW, WH, and YLiu contributed to the collection and assembly of data. ZY and GG contributed to data analysis. All authors contributed to the article and approved the submitted version.

## Funding

This work was supported by grants from the State Key Laboratory of Radiation Medicine and Protection (GZK1201908).

## Conflict of Interest

The authors declare that the research was conducted in the absence of any commercial or financial relationships that could be construed as a potential conflict of interest.
